# Relationship between household food insecurity and food and nutrition literacy among children of 9–12 years of age: a cross-sectional study in a city of Iran

**DOI:** 10.1186/s13104-020-05280-2

**Published:** 2020-09-15

**Authors:** Fatemeh Khorramrouz, Azam Doustmohammadian, Omid Eslami, Majid Khadem-Rezaiyan, Parisa Pourmohammadi, Maryam Amini, Maryam Khosravi

**Affiliations:** 1 Student Research Committee, Department of Nutrition, School of Medicine, Mashhad University of Medical Sciences, Mashhad, Iran; 2 Gastrointestinal and Liver Diseases Research Center, Iran University of Medical Sciences, Tehran, Iran; 3 Department of Nutrition, School of Public Health, Iran University of Medical Sciences, Tehran, Iran; 4 Clinical Research Development Unit of Akbar Hospital, Mashhad University of Medical Sciences, Mashhad, Iran; 5 Department of Nutrition, Varastegan Institute for Medical Sciences, Mashhad, Iran; 6 Department of Nutrition Research, Faculty of Nutrition and Food Technology, National Nutrition and Food Technology Research Institute, Shahid Beheshti University of Medical Sciences, Tehran, Iran; 7 Department of Nutrition, School of Medicine, Mashhad University of Medical Sciences, Vakilabad Blvd, Azadi Square, Mashhad, Iran

**Keywords:** Food and nutrition literacy, Food insecurity, Children

## Abstract

**Objective:**

The aim of this study was to assess the relationship between household food insecurity (HFI) with food and nutrition literacy (FNLIT) in a sample of Iranian children. This cross sectional study was performed on 315 children aged 9 to 12 years recruited from the primary schools throughout Mashhad, Iran. The Household Food Insecurity Access Scale (HFIAS) was used to assess the HFI. Also, overall FNLIT score and its sub-categories were evaluated using a validated 40-item questionnaire.

**Results:**

The prevalence of HFI in the total sample was about 56%. Also, almost 14% of students had a low FNLIT score. Food insecure children had significantly lower levels of FNLT and some subscales including nutrition knowledge, food choice literacy, and food label literacy than the food-secure subjects, moreover, they had a higher likelihood of having low FNLIT score (OR = 2.89, CI 1.03–8.09; p = 0.04). In conclusion, there is a negative association between HFI and FNLIT in children. Further studies to confirm this finding are needed.

## Introduction

Household food insecurity (HFI), which is defined as the limited access to nutritionally adequate and safe food or inability to acquire foods in socially acceptable ways, has become a major public health concern throughout the world. HFI is shown to be positively related to the several adverse health outcomes in children including infectious diseases, anemia, psychological distresses, and growth disorders [1]. Besides the socio-economic, there are some non-financial factors in determining food access at the household level, which are proposed that might influence access to healthy foods in low-income or food-insecure individuals [2, 3]. One of the factors that have been recently gained attention is food and nutrition literacy (FNLIT).

The FNLIT is a relatively new term that reflects the technical, cultural and ethical aspects of the foods beyond just a source of satisfying the caloric requirements [4]. Studies have revealed that food literacy/nutrition literacy can play a critical role in shaping children’s dietary behaviors [5, 6] and enabling them to make healthy food choices that can be sustained later in life [7–9]. Low FNLIT is associated with nutritional inadequacy in school-age children and it seems to be a barrier for them to assess information when choosing foods, comprehend food labels, and apply dietary recommendations [10]. Therefore, identifying the factors that could affect food literacy in children is a necessary approach to promote healthy eating patterns and subsequently, to reduce the burden of diet-related diseases in the long-term.

There is a dual relationship between food security and food literacy whereby inadequate food literacy may contribute to food insecurity and being food insecure may limit the ability to use food literacy skills to achieve adequate diet quality [11]. Some findings have shown that development of food and nutrition literacy components among children including learning about traditional food practices from elders, parents and families members, new food exposure, learning about seasonality and local foods, and food preparation skills as well as food and drink purchasing skills through improving reading and using traffic light and food labels, students grocery store/supermarket tours make meaningful improve to children food choices. This contributes to food utilization as a key dimension of food security [10, 12].

Findings from some recent studies have shown that in comparison to the food-secure households, those with food insecurity had lower FNLIT mainly characterized by unfavorable food choices and purchasing decisions as well as poor food preparation skills [13–16]. However, this evidence was limited to the adult population living in high-income and developed countries; thus, it is unknown whether these findings are generalizable to the children and adolescents particularly in the low-income and developing nations. To our knowledge, no study has examined the relationship between HFI and food literacy in children yet. With this regard, the present study aimed to assess the association of HFI with FNLIT and its sub-categories in a sample of Iranian children.

## Main text

### Methods

A total of 315 children aged 9 to 12 years-old were recruited from the primary schools throughout the city of Mashhad, north-east of Iran. Data were collected from December 2018 to March 2019. Study subjects were chosen using a multi-stage random cluster sampling method. Children in the fourth, fifth and sixth grades who had not any chronic or acute diseases, were eligible to participate in the study.

Sociodemographic data including child’s age, birth order, parents' age, parental education was obtained through interviews with students and verified by their mothers or caregivers by experienced interviewers.

Anthropometric measurements were performed by a trained dietitian using the calibrated equipment. Body mass index (BMI) was calculated as weight in kilograms divided by the square of height in meter. The BMI Z-score for age and sex was calculated based on the World Health Organization Child Growth Standards software (AnthroPlus, World Health Organization, Geneva, Switzerland, 2007). The weight status of children was reported in four categories including underweight (z-score < 2 standard deviation (SD), normal (z-score ≥ – 2 SD and ≤ 1 SD), overweight (z-score > 1 SD and ≤ 2 SD), and obese (z-score > 2 SD).

The Household Food Insecurity Access Scale (HFIAS) was used to assess HFI in the study sample. The validity of the Persian version of questionnaire in the Iranian population was confirmed by Salarkia et al. [17]. The questionnaire consists of 9 items investigating a wide range of food-related behaviors, experiences, and conditions due to the financial limitation over a recall period of past month. Based on the total score, households were categorized as food secure (0–1 point), mild (2–7 points), moderate (8–14 points), and severe food insecure (15–27 points). In this study, mothers or caregivers were interviewed to fill out HFIAS questionnaire. We also have merged the mild, moderate and severe category to the food insecure group as a separate group.

The FNLIT was measured using a developed questionnaire. The questionnaire examining FNLIT in two distinctive domains with seven subscales, including (1) cognition domain: understanding food and nutrition information and nutritional health knowledge; (2) skills domain: functional FNLIT, interactive FNLIT, food choice literacy, critical FNLIT and food label literacy, respectively [18]. We confirmed the validity and reliability of FNLIT in this population. Content Validity Ratio (CVR) and Content Validity Index (CVI) of the 40-item questionnaire were at acceptable levels of 0.87 and 0.99. The internal consistency and test–retest reliability were assessed using Cronbach α (subscale-specific, range: 0.68–0.8) and intra-class correlation coefficients (ICC: 0.97, CI 0.94–98), respectively. FNLIT scores were ranked into three categories as low FNLIT (≤ 58), medium FNLIT (> 58–< 81) and high FNLIT (≥ 81).

#### Statistical analysis

Statistical analysis was performed using SPSS version 25 (SPSS Inc., Chicago, Illinois, USA). Independent-samples t test and Chi-square test was used to compare the variables between the food-secure and food-insecure subjects. Also, to determine the odds of having low FNLIT score in food insecure subjects in comparison to the food secure one, the crude and adjusted multiple regression models were used. The covariates included in the adjusted analyses were sex, grade, BMI, birth order, as well as parental age and education which were stated as the most important socioeconomic predictors of FNLIT in children [19]. Categorical variables were presented as frequency and percentage, while the numerical data were reported as mean, standard deviation (SD), odds ratio (OR), and 95% confidence interval (CI). Significance level was considered as a p-value less than 0.05.

### Results

A total of 315 students (51% males) participated in the study. The mean age of study participants was 10.55 ± 1.007 years. The prevalence of mild, moderate, and severe HFI in the total sample were 30.5%, 20.3%, and 5.7%, respectively. Also, about 14% of the students had a low FNLIT score, while the percentages of moderate and high FNLIT scores in the total study sample were 62.7% and 23.2%, respectively. As it is shown in Table 1, the distribution of BMI, birth order and parental education were significantly different between the food-secure and food-insecure subjects (p < 0.05). Although, there were no significant differences between the two groups in terms of age, sex, grade and parental age.

**Table 1:** General characteristics of study participants

Variable	HFI status		p-value^a^
	Food-secure (n = 137)	Food-insecure (n = 178)	
Age (year)	10.49 ± 1.02^b^	10.58 ± 0.98	0.44^c^
Sex			
Male	73 (45.1)^c^	89 (54.9)	0.56
Female	64 (41.8)	89 (58.2)	
Grade			
Fourth	50 (46.3)	58 (53.7)	0.73
Fifth	43 (41.0)	62 (59.0)	
Sixth	44 (43.1)	58 (56.9)	
BMI z-score			
Underweight	8 (40.0)	12 (60.0)	0.02*
Normal	76 (42.0)	105 (58.0)	
Overweight	20 (33.3)	40 (66.7)	
Obese	33 (61.1)	21 (38.9)	
Birth order			
1	81 (54.0)	69 (46.0)	< 0.001*
≥ 2	56 (33.9)	109 (66.1)	
Father age tertile (year)			
30–37	31 (40.8)	45 (59.2)	0.15
38–44	69 (49.6)	70 (50.4)	
≥ 45	36 (37.5)	60 (62.5)	
Mother age tertile (year)			
23–34	43 (45.7)	51 (54.3)	0.56
35–39	57 (46.0)	67 (54.0)	
≥ 40	37 (39.4)	57 (60.6)	
Father education			
≤ 5 years education	2 (4.9)	39 (95.1)	< 0.001*
6 to 9 years or diploma	37 (26.4)	103 (73.6)	
Associate’s degree and higher	97 (75.2)	32 (24.8)	
Mother education			
≤ 5 years education	1 (3.4)	28 (96.6)	< 0.001*
6–9 years or diploma	38 (23.3)	125 (76.7)	
Associate’s degree and higher	98 (81.7)	22 (18.3)	

*HFI* household food insecurity, *BMI* body mass index

*Significant at the level of p < 0.05

^a^ p-values obtained from Chi-square test unless indicated

^b^ Data are shown as Mean ± standard deviation

^c^ p-value obtained from Independent-samples t test

^c^ Data are shown as frequency (percentage)

Table 2 compares the total FNLIT scores and its subscales between the food-secure and food-insecure children. In comparison to the food-insecure group, the food-secure subjects had significantly higher scores for total FNLIT (p < 0.001) and some subscales including understanding food and nutrition information (p = 0.01), nutritional health knowledge (p = 0.001) as well as food choice literacy (p = 0.009) and food label literacy (p < 0.001). However, the mean scores of functional, interactive, and critical FNLIT subscales were not significantly different between the two groups.

**Table 2:** Comparison of mean scores of total FNLIT and its subscales based on the HFI status

Variable	HFI status		p-value^a^
	Food-secure (n = 137)	Food-insecure (n = 178)	
Total FNLIT (score)	74.09 ± 11.57^b^	68.65 ± 12.83	< 0.001*
FNLIT cognitive domain (score)			
Understanding food and nutrition information	76.95 ± 12.39	73.25 ± 13.91	0.01*
Nutritional health knowledge	88.5 ± 13.49	82.12 ± 20.74	0.001*
FNLIT skill domain (score)			
Functional FNLIT	72.03 ± 13.9	69.41 ± 16.13	0.12
Interactive FNLIT	57.05 ± 26.9	57.11 ± 24.86	0.98
Food choice literacy	75.86 ± 69.94	69.94 ± 21.97	0.009*
Critical FNLIT	67.88 ± 22.16	65.87 ± 23.75	0.43
Food label literacy	72.08 ± 29.7	49.01 ± 35.19	< 0.001*

*HFI* household food insecurity, *FNLIT* food and nutrition literacy

*Significant at the level of p < 0.05

^a^ p-values obtained from Independent-samples t test

^b^ Data are shown as Mean ± standard deviation

The results of linear regression models of the influence of HFI on changes in overall FNLIT score and its sub-categories are presented in (Additional file 1: Table S1). Based on the results of logistic regression analysis shown in Table 3, the food-insecure group had a higher likelihood of having low FNLIT compared to the food–secure group (OR = 2.86, 95% CI 1.35, 6.05; p = 0.006). Also, this association remained significant after adjusting for potential covariates (OR = 2.89, 95% CI 1.03, 8.09; p = 0.04).

**Table 3:** Crude and adjusted odds of having low FNLIT according to the HFI status obtained from logistic regression analysis (n = 315)

	Crude analysis		Adjusted analysis^a^	
	Low FNLIT		Low FNLIT	
	OR (95% CI)	p-value	OR (95% CI)	p-value
HFI status				
Food secure	Reference		Reference	
Food insecure	2.86 (1.35–6.05)	0.006*	2.89 (1.03–8.09)	0.04*

*FNLIT* food and nutrition literacy, *OR* odds ratio, *CI* confidence interval, *HFI *household food insecurity

*Significant at the level of p < 0.05

^a^ Adjusted for sex, grade, BMI, birth order, parental age and education

### Discussion

To our knowledge, this is the first study investigating the association between HFI and FNLIT in a sample of school-aged children in Iran. We found that children living in the food-insecure households had higher odds of having overall FNLIT compared to the food-secure children. Also, HFI was associated with poor FNLIT behaviors including nutritional knowledge, food choice literacy, and food label literacy.

Findings of the present study are in consistent with the earlier studies, mainly performed in the adults’ population. In a study by Begley et al. among a sample of Australian adults, all domains of the Australian food literacy model including planning and management, shopping, preparation, and cooking were independently associated with food insecurity [13]. Also, in another study among college students, it was found that very low food secure students had significantly lower cooking self-efficacy and food preparation scores compared with food secure students [15]. Similarly, Mercille et al. demonstrated that severe household food insecurity was inversely associated with healthy food preparation and self-efficacy among Canadian aboriginal women [20]. Despite the consistent results, there have been a considerable differences between the above-mentioned studies in terms of the type of measurement tools for assessment of food insecurity and different aspects of FNLT. Also, all the previous studies were conducted in the adults from developed countries that should be considered when the interpretation of the results.

The findings indicate students experiencing HFI had a lower level of nutritional knowledge and were less able to understand food and nutrition information. Also, the food choice literacy scores was lower in the food-insecure subjects. This result is consistent with many studies demonstrated HFI are associated with unfavorable eating behaviors and consequently low dietary quality, particularly among children [21–24]. Landry et al. reported food insecure children had poor diet quality and lower scores for greens and beans, seafood and plant proteins, and added sugar compared to the food-secure ones [21]. Similarly, in a study conducted on 3790 food-insufficient, low-income families, children consumed fewer calories, total carbohydrates and fruits, but higher cholesterol intake [22].

Moreover, children experiencing different degrees of food insecurity had higher tendency to purchase foods from stores that contain less-healthful foods particularly the convenience ones, and also making unfavorable food choices such as purchasing unhealthy snack foods and sugar sweetened beverages [25, 26]. Although food literacy as a key component of shaping eating behaviors can improve food choices by helping food insecure children to develop their skills, many unmodifiable factors contribute to make poor food choices such as household income level, poor food access and availability and the high prices of healthy food.

The results also suggest that HFI was associated with lower level of food label literacy. Consistent with our observation Butcher et al. found Australian households with low or very low HFI status less tend to find, use or be influenced by nutrition information on food labels when making dietary decisions [14]. Similarly, Gittelsohn et al. reported the lowest food label reading and food knowledge scores among food-insecure households in Baltimore City in the USA [16]. Although there is a growing body of evidence suggested that food label literacy may lead to healthier food purchases [27, 28], the less use of food labels in food-insecure households probably due to families' priorities. In confronting with financial constraints, food purchasing decisions of families are based on price and family food preferences rather than nutrition considerations [29].

## Conclusion

In summary, we found that HFI is associated with children's food and nutrition literacy. Food insecure children had lower levels of FNLIT and its subscales including nutrition knowledge, food choice literacy, and food label literacy. We conclude that HFI may be one of the predictors of low FNLIT in primary school children. However, more research is needed to clarify the relationship between food security and food literacy in children to inform Policymaking (Additional file 1: Table S1).

## Limitations

There are a few limitations that need to be acknowledged. This is a cross-sectional study that can only establish an association between HFI and food literacy and cannot show causality. Also, the study was conducted in a small group of children aged 9 to 12 years-old in north-east of Iran; therefore, the generalizability of findings to the Iranian populations and different age groups needs to be considered.

## Supplementary information

**Additional file 1: Table S1.** Crude and adjusted association between HFI and total FNLIT scores and its subscales obtained from multiple linear regression analysis.

## Data Availability

The datasets used and/or analyzed during the current study are available from the corresponding author on reasonable request.

## Abbreviations

HFI: Household food insecurity
FNLIT: Food and nutrition literacy
BMI: Body mass index
SD: Standard deviation
HFIAS: Household Food Insecurity Access Scale
CVR: Content Validity Ratio
CVI: Content Validity Index
ICC: Interclass coefficients
OR: Odds ratio
CI: Confidence interval
