# Supplementary material for: Relationship between household food insecurity and food and nutrition literacy among children of 9–12 years of age: a cross-sectional study in a city of Iran
Source: BMC Res Notes. 2020 Sep 15;13:433. doi: 10.1186/s13104-020-05280-2 (PMC7493354; doi:10.1186/s13104-020-05280-2)
Supplement: Supplementary file 1 — Additional file 1: Table S1. Crude and adjusted association between HFI and total FNLIT scores and its subscales obtained from multiple linear regression analysis. [file 13104_2020_5280_MOESM1_ESM.docx]

Table S1. Crude and adjusted association between HFI and total FNLIT scores and its subscales obtained from multiple linear regression analysis.

| Variable | Crude analysis | | Adjusted analysis ^a^ | |
| --- | --- | --- | --- | --- |
|  | β | p-value | β | p-value |
| Total FNLIT score | - 0.29 | <0.001* | - 0.12 | 0.006* |
| Understanding of food and nutrition information | - 0.20 | <0.001* | - 0.08 | 0.05 |
| nutritional health knowledge | - 0.22 | <0.001* | - 0.11 | 0.01* |
| functional FNLIT | - 0.12 | 0.03* | - 0.03 | 0.49 |
| interactive FNLIT | - 0.09 | 0.09 | - 0.05 | 0.21 |
| food choice literacy | - 0.19 | 0.001* | - 0.06 | 0.13 |
| critical FNLIT | - 0.07 | 0.2 | - 0.01 | 0.73 |
| Food label literacy | - 0.36 | <0.001* | - 0.15 | <0.001* |

^a^ Adjusted for sex, grade, BMI, birth order, parental age and education.

Significant at the level of p< 0.05*
